# Efficacy and safety of combined targeted therapy and immunotherapy versus targeted monotherapy in older patients with uHCC

**DOI:** 10.3389/fonc.2025.1515640

**Published:** 2025-04-28

**Authors:** Yu Li, Jian-Hong Zhong, Xiao-Dong Zhu, Chuang-Ye Han, Jia-Bei Wang, Hong-Zhi Liu, Kuan Hu, Yang-Xun Pan, Hui-Chuan Sun, Tao Peng, Lian-Xin Liu, Yong-Yi Zeng, Le-Du Zhou, Li Xu, Nan-Ya Wang

**Affiliations:** ^1^ Department of Phase I Clinical Trial Center, The First Hospital of Jilin University, Changchun, Jilin, China; ^2^ Department of Hepatobiliary Surgery, The Affiliated Cancer Hospital of Guangxi Medical University, Nanning, Guangxi, China; ^3^ Department of Liver Surgery and Transplantation, Liver Cancer Institute and Zhongshan Hospital, Fudan University, Shanghai, China; ^4^ Department of Hepatobiliary Surgery, The First Affiliated Hospital of Guangxi Medical University, Nanning, Guangxi, China; ^5^ Department of Hepatobiliary Surgery, The First Affiliated Hospital of University of Science and Technology of China (USTC), Hefei, Anhui, China; ^6^ Department of Hepatobiliary Surgery, Mengchao Hepatobiliary Hospital of Fujian Medical University, Fuzhou, Fujian, China; ^7^ Department of Hepatobiliary Surgery, Xiangya Hospital, Central South University, Changsha, Hunan, China; ^8^ Department of Liver Surgery, Sun Yat-Sen University Cancer Center, Sun Yat-Sen University, Guangzhou, Guangdong, China

**Keywords:** hepatocellular carcinoma, older patients, targeted monotherapy, PD-1/PD-L1 combination therapy, clinical outcome

## Abstract

**Background:**

The prevalence of hepatocellular carcinoma (HCC) among older patients is rising due to the aging population. This study aimed to compare the efficacy and safety of targeted therapy alone versus its combination with immunotherapy in older patients (≥ 65 years old) with unresectable HCC (uHCC).

**Methods:**

We retrospectively analyzed 158 patients aged ≥ 65 diagnosed with uHCC who received targeted therapy alone or in combination with immunotherapy from the CLEAP database between March 2019 and July 2023. The primary endpoint was overall survival (OS), with secondary endpoints including progression-free survival (PFS), objective response rate (ORR), disease control rate (DCR), and safety assessments for adverse events (AEs).

**Results:**

The ORR was 3.6% in the targeted monotherapy group compared to 29.4% in the combination therapy group, while the DCRs were 53.6% and 54.9%, respectively. Survival analysis indicated a median PFS of 7.3 months for monotherapy versus 13.2 months for combination therapy (*P* = 0.137) and a median OS of 16.0 months versus 20.0 months, respectively (*P* = 0.140). AEs occurred in 44.6% of the monotherapy group and 58.8% in the combination therapy group, with 20.5% in the combination group withdrawing due to adverse reactions, significantly higher than in monotherapy group.

**Conclusion:**

Among older patients with uHCC, the combination therapy demonstrated higher ORR and longer PFS and OS, although it had higher incidences of AEs and drug withdrawal.

## 1 Introduction

Hepatocellular carcinoma (HCC) is the sixth most common malignant tumor and the third leading cause of cancer-related deaths worldwide (1). With an aging population, the incidence of HCC among older individuals has been steadily rising (2, 3). China has a high incidence of HCC and a serious aging population (4). The incidence and mortality rates of HCC in people over 65 years old are 8.1% and 9.7%, respectively, which are higher than the global average (5–7). By 2028, an estimated 21.3% of patients with HCC will be over 80 years old (8).Surgical treatments are often unsuitable for many older patients at diagnosis, making systemic treatment the primary approach.

The first-line systemic therapy for HCC mainly includes targeted therapy, immunotherapy and immune combination therapy. The NCCN, ASCO, ESMO, AASLD, and CSCO guidelines all recommend sorafenib, lenvatinib, atezolizumab plus bevacizumab, etc. as the preferred first-line treatment for HCC (9–12). Since 2008, sorafenib has opened a new era of targeted therapy for HCC with two large-scale, randomized controlled international multi-center clinical trials, SHARP and Oriental (13, 14). Subsequently, lenvatinib has also shown good efficacy (15, 16). In 2020, a landmark phase III clinical trial IMbrave150 proved that combined immunotherapy is superior to targeted monotherapy, the treatment of HCC has entered a new era of targeted combined immunotherapy (17). Since then, two other phase III clinical trials have also confirmed that compared with targeted monotherapy, combined targeted therapy and immunotherapy has significant survival benefit (18, 19). However, current studies often enroll a limited number of older patients, leading to significant underrepresentation. This is frequently due to the exclusion of older adults with complex comorbidities, which limits the generalizability of the findings. As a result, the existing evidence may not fully capture the real-world clinical outcomes, safety profiles, or therapeutic responses of interventions in this vulnerable population. It is still necessary to explore whether elderly uHCC patients are more suitable for targeted monotherapy or combined targeted therapy and immunotherapy (the following use combination immunotherapy to refer).

Therefore, our study aimed to comprehensively compare the efficacy and safety of applying targeted monotherapy with the combination immunotherapy in elderly uHCC patients by using a multicenter database, and provide a safer and more effective decision-making for elderly patients.

## 2 Patients and methods

### 2.1 Patient selection

We conducted this multicenter study between March 2019 and July 2023 in eight hospitals. All data are from the CLEAP database. We conducted a retrospective analysis of clinical data from patients aged ≥65 years diagnosed with uHCC during this period, who received targeted monotherapy (including, but not limited to, sorafenib or lenvatinib) or combined targeted therapy (including sorafenib, lenvatinib, bevacizumab, and others) and immunotherapy (PD-1/PD-L1 inhibitors such as pembrolizumab, atezolizumab, etc.). The study was conducted in accordance with the Declaration of Helsinki and the Ethical Guidelines for Clinical Studies. The study was approved by the Institutional Review Board of the China Liver Cancer Study Group Young Investigators (CLEAP) (B2022-195R). And the participants provided their written informed consent to participate in this study.

**Figure 1:**
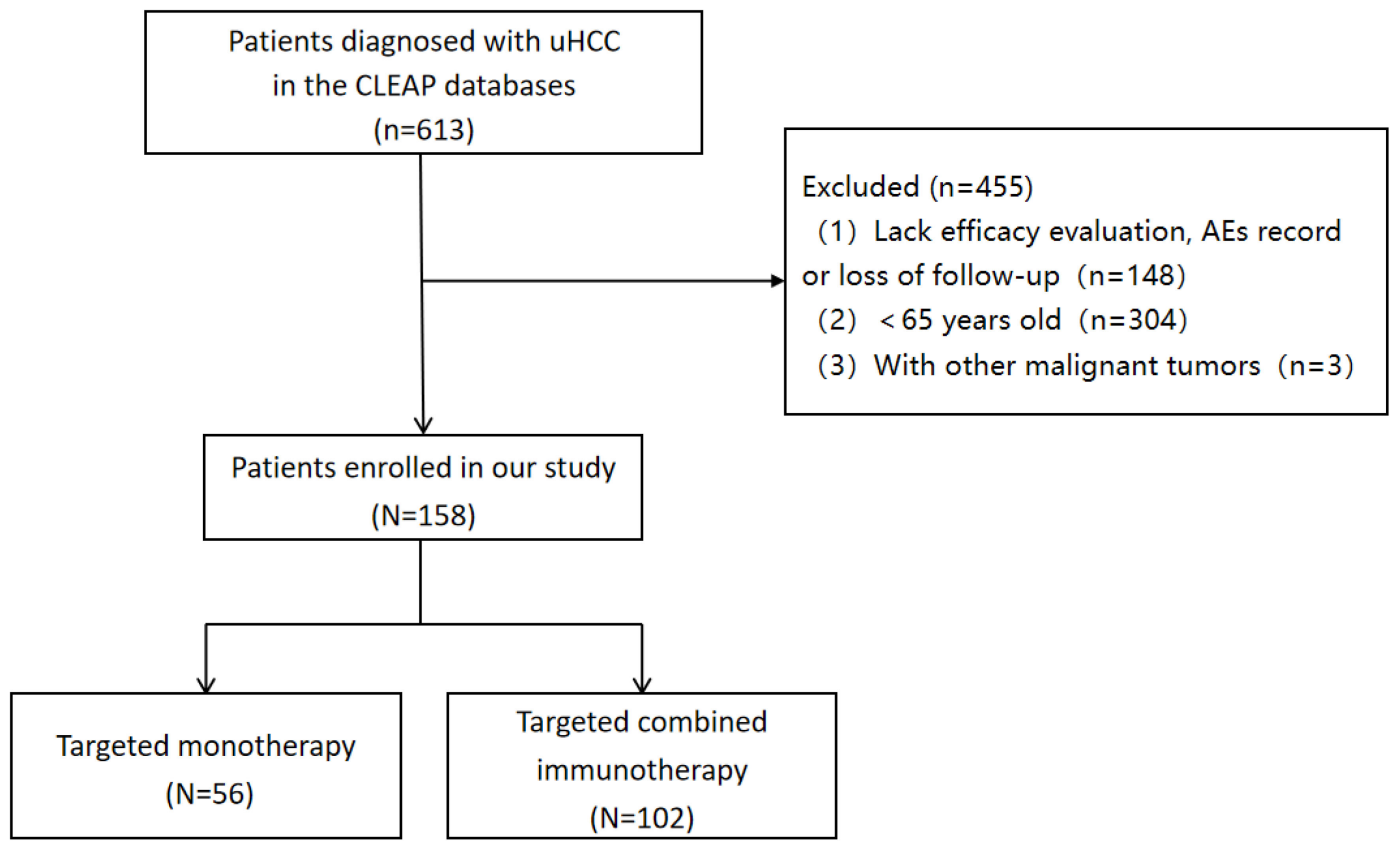
Flow chart of the cohort.

Inclusion criteria were: (1) patients aged ≥ 65 years with a clinical imaging or pathological diagnosis of HCC; (2) patients with BCLC stages B or C confirmed by imaging; (3) patients receiving targeted immunotherapy or monotherapy; (4) at least one imaging assessment for treatment efficacy. Exclusion criteria were: (1) patients aged < 65 with clinical imaging or diagnosis of HCC; (2) Lack efficacy evaluation, AEs record or loss of follow-up; and (3) presence of other malignant tumors (**Figure 1**).

### 2.2 Treatment

Patients in the targeted monotherapy group received targeted therapies such as sorafenib or lenvatinib, which is based on weight. The combination immunotherapy group received PD-1/PD-L1 inhibitors (such as pembrolizumab,atezolizumab, etc.) in combination with targeted therapies such as sorafenib, lenvatinib, or bevacizumab. The above drug doses are used according to the recommended dose.

### 2.3 Patient follow-up and clinical outcomes

The first follow-up evaluation was performed 4-6 weeks after treatment. After the first follow-up, the strategies were performed every 2-3 months. The follow-up contents mainly included laboratory indicators (such as alpha-fetoprotein levels, blood counts, liver and kidney function, etc.) and imaging examination (such as lung CT and abdominal CT/MRI). Tumor response was evaluated using RECIST 1.1 criteria. Patient ‘s AEs were asked and recorded in the medical case during each diagnosis and treatment. Patients who did not attend the clinic were asked and collected AEs by telephone follow-up. Adverse drug reactions were classified using Common Terminology Criteria for Adverse Events (CTCAE 5.0) criteria. All patients were followed up until death, loss to follow-up, or December 2023. The primary endpoint was overall survival (OS), and secondary endpoints were progression-free survival (PFS), objective response rate (ORR), and disease control rate (DCR).

### 2.4 Statistical analysis

Statistical analyses were conducted using SPSS 26.0 and R. Measurement data were expressed as $\bar{\text{x}}$ ± s, and the t-tests were used for group comparisons. The count data were expressed as the number of cases (percentage), and the X^2^ test was used for comparison between the two groups. The survival time between the two groups was analyzed using the Kaplan-Meier survival curve, with log-rank tests to assess the differences. Univariate and multivariate Cox regression analyses were conducted to determine risk factors affecting survival, including variables, with *P* < 0.05 from univariate analyses in the multivariate model. *P* < 0.05 was considered statistically significant.

## 3 Results

### 3.1 Baseline clinical characteristics

A total of 158 eligible patients were consecutively enrolled in our study, with 56 receiving targeted monotherapy (including 49 cases of Lenvatinib, 4 cases of donafenib, and 3 cases of sorafenib) and 102 receiving combination immunotherapy (including 6 cases of atezolizumab, 4 cases of pembrolizumab, and 92 cases of others, such as sintilimab, tislelizumab, toripalimab and camrelizumab). The median age of all patients was 68 years (65–85 years), and 76.6% were male. Among the participants, 122 patients (77.2%) had hepatitis virus infection, 106 (67.1%) had cirrhosis, 76 (48.1%) had portal vein tumor thrombus, and 58 (36.7%) had ascites. Among all the patients, 112 (79.9%) received local treatment (TACE or HAIC). In the targeted monotherapy group, the proportion of patients who received local treatment was significantly lower than in the combination immunotherapy group (60.7% vs. 76.5%, *P* = 0.037). Similarly, the percentage of patients classified as Child-Pugh A was also lower in the monotherapy group compared to the combination group (39.3% vs. 70.6%, *P* = 0.001). No significant differences in demographic or tumor characteristics were observed between the two groups, aside from local treatment and Child-Pugh scores (*P* > 0.05) (**Table 1**).

**Table 1:** Baseline clinical characteristics of elderly patients with uHCC.

Baseline data	Targeted monotherapy N=56	Targeted combined immunotherapy N=102	*P*
Sex			0.728
male Female	42 (75.0%) 14 (25.0%)	79 (77.5%) 23 (22.5%)	
Hepatitis			0.464
No HBV HCV	11 (19.6%) 32 (57.1%) 13 (23.2%)	25 (24.5%) 61 (59.8%) 16 (15.7%)	
Antiviral drug			0.478
No Yes	28 (50.0%) 28 (50.0%)	45 (44.1%) 57 (55.9%)	
ECOG-PS			0.067
0 1 2	21 (37.5%) 29 (51.8%) 6 (10.7%)	55 (53.9%) 43 (42.2%) 4 (3.9%)	
Individual history			
smoking drinking hypertension diabetes coronary heart disease renal insufficiency cercbral disease	15 (26.8%) 15 (26.8%) 18 (32.1%) 15 (26.8%) 8 (14.3%) 7 (12.5%) 9 (16.1%)	18 (17.6%) 19 (18.6%) 42 (41.2%) 24 (23.5%) 18 (17.6%) 8 (7.8%) 9 (8.8%)	0.176 0.233 0.263 0.650 0.586 0.339 0.170
Cirrhosis			0.055
No Yes	13 (23.2%) 43 (76.8%)	39 (38.2%) 63 (61.5%)	
Portal vein tumor thrombus			0.100
No Yes	34 (60.7%) 22 (39.3%)	54 (47.1%) 48 (52.9%)	
Ascites			0.060
No Yes	30 (53.6%) 26 (46.4%)	70 (68.6%) 32 (31.4%)	
Distant metastasis			0.669
No Yes	36 (64.3%) 20 (35.7%)	69 (67.6%) 31 (32.4%)	
AFP			0.067
<400 ≥ 400	37 (66.1%) 19 (33.9%)	52 (51.0%) 50 (19.0%)	
Child-Pugh			0.001
A B C	22 (39.3%) 32 (57.1%) 2 (3.6%)	72 (70.6%) 29 (28.4%) 1 (1.0%)	
BCLC			0.820
B C	18 (32.1%) 38 (67.9%)	31 (30.4%) 71 (69.6%)	
Receive topical treatment during medication (TACE or HAIC)			0.037
No Yes	22 (39.3%) 34 (60.7%)	24 (23.5%) 78 (76.5%)	

### 3.2 Efficacy and survival

**Figure 2:**
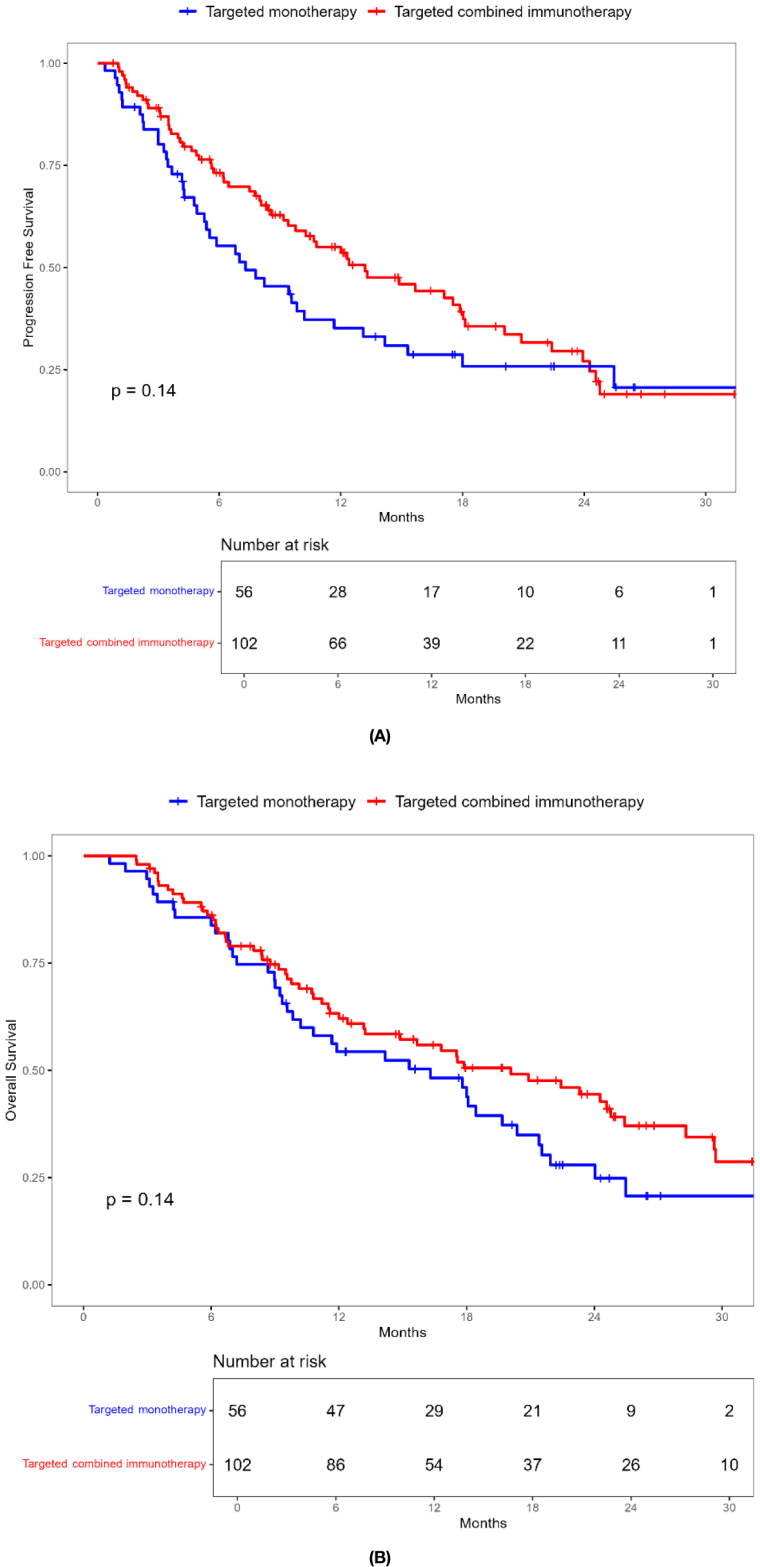
Kaplan-Meier survival curves of progression-free survival **(A)** and overall survival **(B)** of elderly uHCC patients treated with targeted monotherapy and targeted combined immunotherapy in the entire cohort.

During a median follow-up of 17.8 months (1.23-52.20 months), a total of 99 patients (62.6%) died. The ORR in the combination immunotherapy group was significantly higher than in the targeted monotherapy group (29.4% vs. 3.6%, *P* = 0.001). DCR was 54.9% for the combination immunotherapy group and 53.6% for the monotherapy group, with no significant difference (**Table 2**). Median PFS (13.2 vs. 7.3 months, *P* = 0.137) and OS (20.0 vs. 16.0 months, *P* = 0.140) were longer in the combination immunotherapy group compared with the targeted monotherapy group (**Figure 2**, **Table 3**). In the monotherapy group, the 6-month, 12-month, and 24-month PFS rates were 55.3%, 35.2%, and 20.7%, respectively, while OS rates were 83.8%, 54.4%, and 24.8%, respectively. In the combination immunotherapy group, the corresponding rates were 72.0%, 53.6%, and 24.6% for PFS and 85.1%, 62.1%, and 42.7% for OS, respectively.

**Table 2:** Comparison of efficacy in elderly patients with uHCC.

	Targeted monotherapy N=56	Targeted combined immunotherapy N=102	*P*
Response			
CR	0 (0.0%)	7 (6.9%)	
PR	2 (3.6%)	23(22.5%)	
SD	28(50.0%)	26(25.5%)	
PD	26(46.4%)	46(45.1%)	
ORR, %	3.6%	29.4%	0.001
DCR, %	53.6%	54.9%	0.872

**Table 3:** PFS and OS of elderly patients with uHCC in the targeted monotherapy and the targeted combined immunotherapy group.

	Targeted monotherapy N=56	Targeted combined immunotherapy N=102	*P*
PFS,median (95% CI) months	7.3	13.2	0.137
6- months PFS rate,% 12-months PFS rate,% 24-months PFS rate,%	55.3% 35.2% 20.7%	72.0% 53.6% 24.6%	
OS,median (95% CI) months	16.0	20.0	0.140
6- months OS rate,% 12-months OS rate,% 24-months OS rate,%	83.8% 54.4% 24.8%	85.1% 62.1% 42.7%	

### 3.3 Subgroup analysis

**Figure 3:**
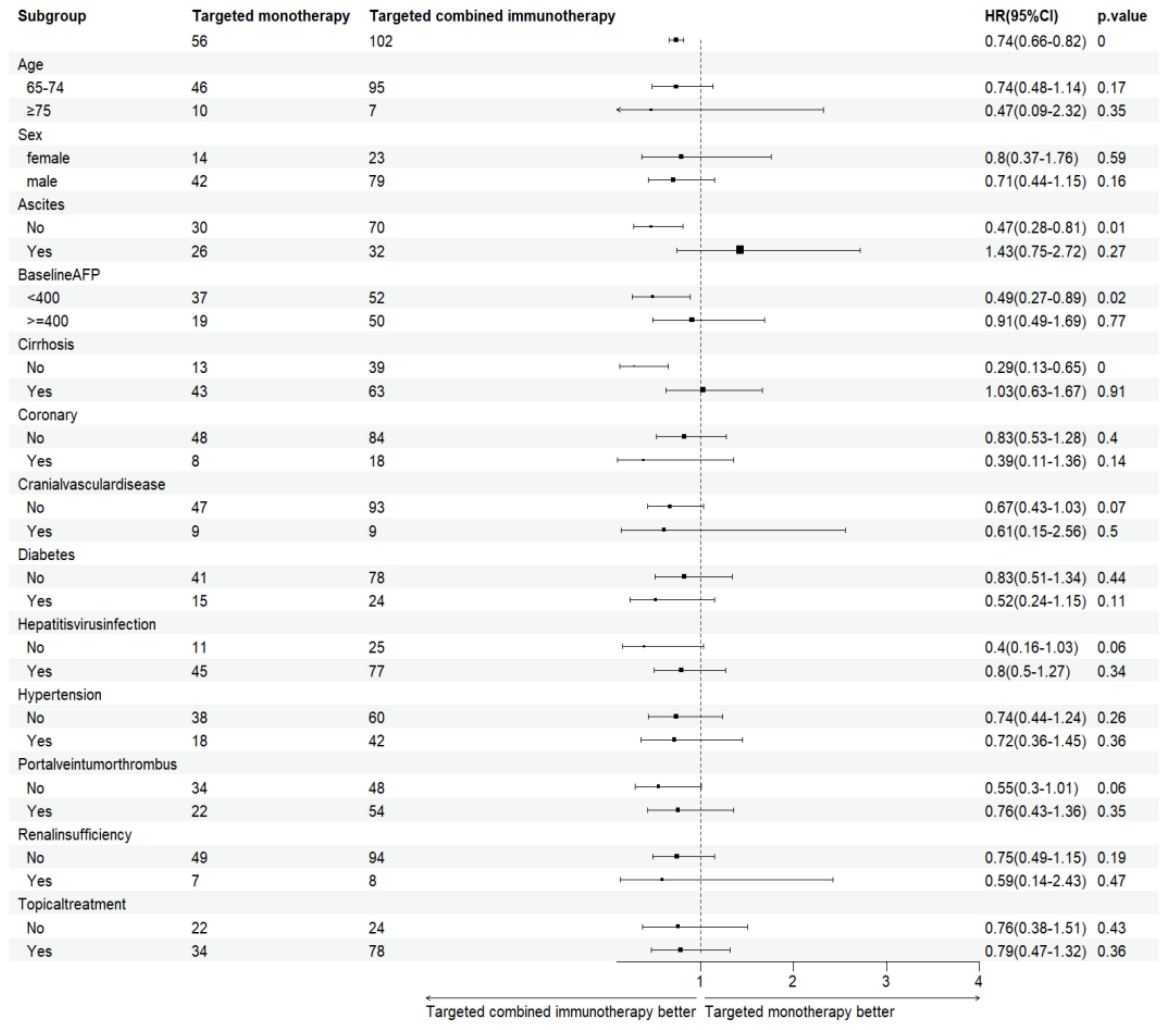
Subgroup analysis of elderly patients with uHCC in the targeted monotherapy and the targeted combined immunotherapy group. P < 0.05, the difference was statistically significant. HR, Hazard Ratio; AFP, alpha-fetoprotein.

**Figure 4:**
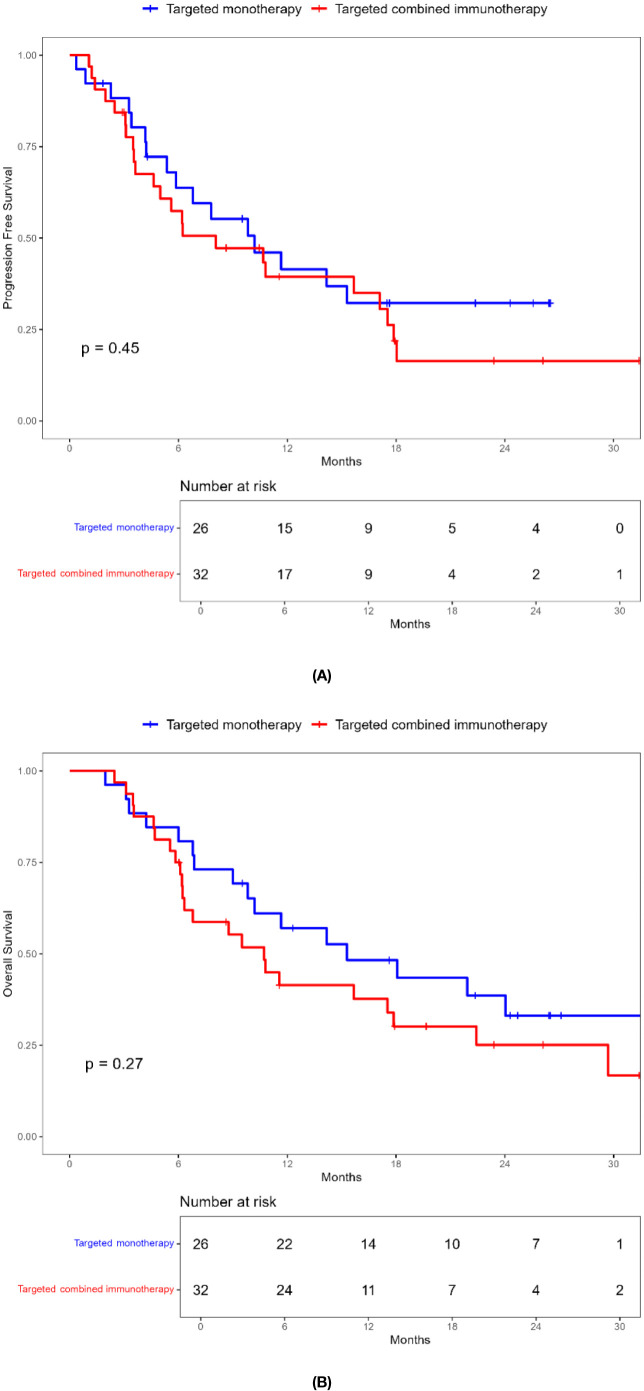
Kaplan-Meier survival curves of progression-free survival **(A)** and overall survival **(B)** of elderly uHCC patients without ascites in the targeted monotherapy and the targeted combined immunotherapy group.

**Figure 5:**
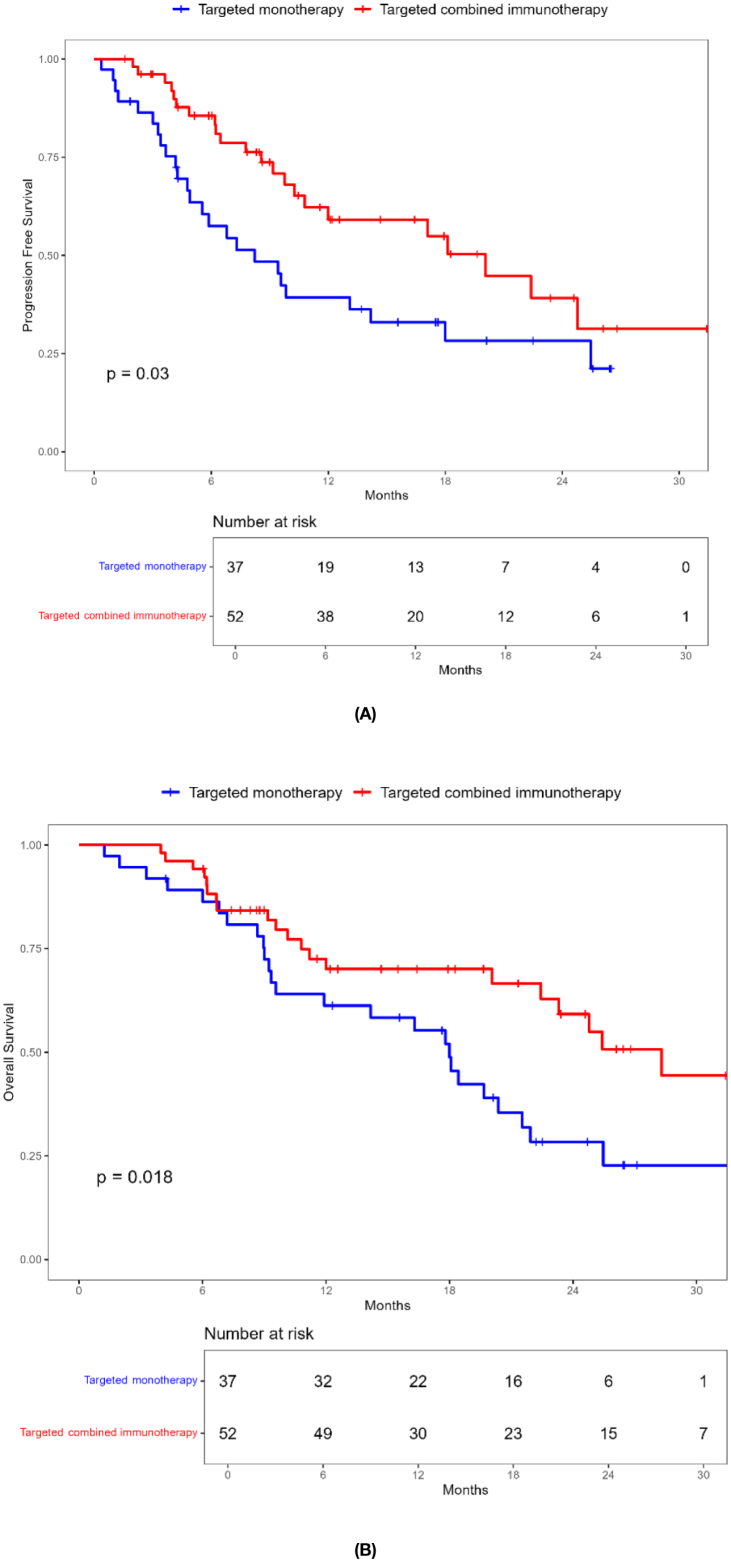
Kaplan-Meier survival curves of progression-free survival **(A)** and overall survival **(B)** of elderly uHCC patients with baseline AFP < 400ng / mL in the targeted monotherapy and the targeted combined immunotherapy group.

**Figure 6:**
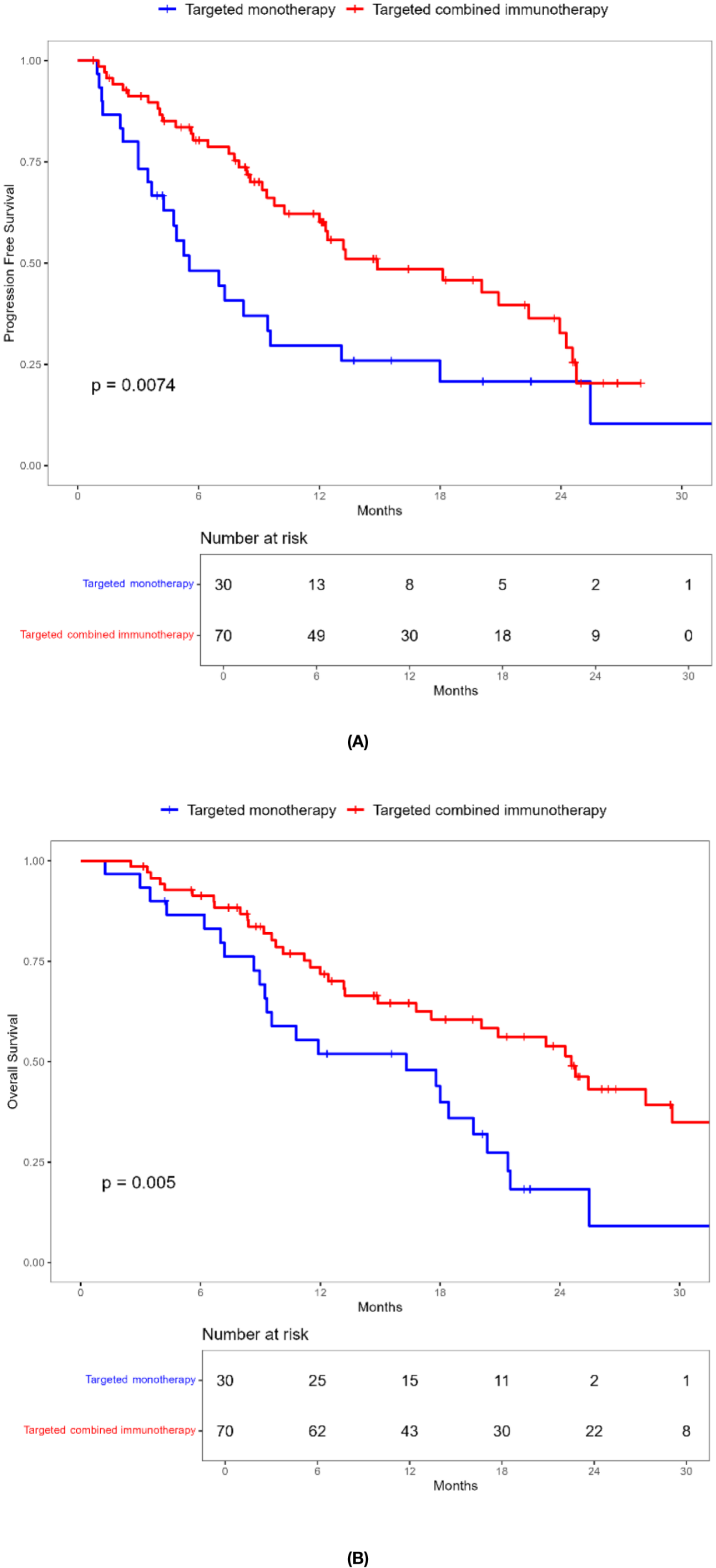
Kaplan-Meier survival curves of progression-free survival **(A)** and overall survival **(B)** of elderly uHCC patients with ascites in the targeted monotherapy and the targeted combined immunotherapy group.

Combination immunotherapy therapy showed greater benefits than targeted monotherapy in most subgroups (**Figure 3**). Patients without ascites and with baseline AFP < 400 ng/mL had significantly improved PFS and OS with combination immunotherapy, and the difference was statistically significant (**Figures 4**, **5**). However, patients with ascites had longer PFS (10.2 vs. 8.0 months, *P* = 0.446) and OS (15.0 vs. 10.0 months, *P* = 0.271) in the targeted monotherapy group (**Figure 6**).

### 3.4 Analysis of prognostic factors

Prognostic factors affecting OS were identified using Cox regression analysis. In univariate analysis, the risk factors in older patients included BCLC (HR: 1.778, 95% *CI*: 1.121-2.819, *P* = 0.014), portal vein tumor thrombus(HR: 1.990, 95% *CI*: 1.334-2.967, *P* = 0.001), cerebrovascular disease (HR: 0.480, 95% *CI*: 0.232-0.991, *P* = 0.047), previous surgical treatment(HR: 0.424, 95% *CI*: 0.219-0.821, *P* = 0.011), elevated glutamic oxaloacetic transaminase (AST) (HR: 1.007, 95% *CI*: 1.002-1.011, *P* = 0.003), and AFP ≥ 400 ng/mL (HR: 1.774, 95% *CI*: 1.194-2.637, *P* = 0.005). After incorporating meaningful variables into multivariate analysis, cerebrovascular disease (HR: 0.467, 95% *CI*: 0.220-0.992, *P* = 0.048) and previous surgical treatment (HR: 0.500, 95% *CI*: 0.252-0.994, *P* = 0.048) were the risk factors independently associated with prognosis in older patients with uHCC (**Table 4**).

**Table 4:** Analysis of univariate and multivariate factors affecting the OS of elderly patients with uHCC.

	Univariate analysis			Multivariate analysis		
	HR	95%CI	*P*	HR	95%CI	*P*
Sex	1.037	0.663-1.622	0.874			
Hepatitis	0.791	0.582-1.075	0.134			
Antiviral drug	0.738	0.496-1.098	0.134			
Child-Pugh	1.363	0.942-1.973	0.100			
ECOG-PS	1.048	0.771-1.424	0.765			
BCLC	1.778	1.121-2.819	0.014	1.407	0.819-2.240	0.216
Distant metastasis	1.151	0.765-1.735	0.500			
Portal vein tumor thrombus	1.990	1.334-2.967	0.001	1.379	0.819-2.321	0.226
Ascites	1.369	0.914-2.051	0.128			
Cirrhosis	1.509	0.690-1.627	0.792			
Hypertension	1.122	0.750-1.678	0.575			
Diabetes	1.188	0.762-1.854	0.447			
Coronary heart disease	0.626	0.363-1.079	0.092			
Renal insufficiency	0.926	0.466-1.840	0.827			
Cercbral disease	0.480	0.232-0.991	0.047	0.467	0.220-0992	0.048
Smoking	1.307	0.811-2.105	0.272			
Drinking	1.243	0.778-1.986	0.362			
Targeted combined immunotherapy	0.736	0.489-1.108	0.142			
Received surgical treatment	0.424	0.219-0.821	0.011	0.500	0.252-0.994	0.048
Receive radiotherapy	1.311	0.699-2.458	0.398			
Receive topical treatment	0.753	0.495-1.145	0.184			
AFP	1.774	1.194-2.637	0.005	1.380	0.867-2.222	0.172
WBC	1.019	0.941-1.104	0.638			
AST	1.007	1.002-1.011	0.003	1.003	0.998-1.008	0.189
ALT	1.001	0.997-1.005	0.693			
TBil	1.007	0.998-1.016	0.113			
ALB	0.967	0.932-1.002	0.063			
PT	1.075	0.997-1.182	0.141			

### 3.5 Safety analysis

A total of 12 patients without AEs records were excluded. Among the 158 patients included, all were systematically assessed and monitored for AEs. A total of 85 patients (53.7%) had AEs and the rest clearly recorded that no AEs occurred. The incidence of AEs in the targeted monotherapy group was 44.6%, with abdominal distension (16.6%), fatigue (14.2%), and decreased appetite (10.7%) as the most common reactions. The combination immunotherapy group had a higher AE rate of 58.8%, with fatigue (17.6%), abdominal distension (13.7%), and hypertension (12.7%) being the most prevalent (**Table 5**). In the general population, 29 patients (18.4%) discontinued therapy, 26 patients (89.7%) due to AE, and 3 patients (10.3%) due to personal reasons. The rate of discontinuation due to AEs was significantly higher in the combination immunotherapy group than the targeted monotherapy group, and the difference was statistically significant (20.5% vs. 8.9%, *P* = 0.005).

**Table 5:** Incidence of AEs in elderly patients with uHCC in the targeted monotherapy and the targeted combined immunotherapy group.

AE, n (%)	Targeted monotherapy N=56	Targeted combined immunotherapy N=102	*P*
**Incidence of AEs**	25 (44.6%)	60 (58.8%)	0.087
**Fatigue**	**8 (14.2%)**	**18 (17.6%)**	0.586
**Dizziness**	0 (0%)	5 (4.9%)	0.092
**Headache**	0 (0%)	3 (2.9%)	0.195
**Diarrhea**	5 (8.9%)	6 (5.8%)	0.472
**Abdominal pain**	2 (3.5%)	11 (10.7%)	0.115
**Abdominal distension**	**9 (16.6%)**	**14 (13.7%)**	0.689
**Nausea**	4 (7.1%)	11 (10.7%)	0.455
**Vomiting**	2 (3.5%)	11 (10.7%)	0.115
**Appetite loss**	6 (10.7%)	12 (11.7%)	0.842
**Weight loss**	2 (3.5%)	3 (2.9%)	0.829
**Hypertension**	3 (5.3%)	**13 (12.7%)**	0.141
**Proteinuria**	4 (7.1%)	8 (7.8%)	0.874
**Constipation**	0 (0%)	4 (3.9%)	0.133
**Hand-foot skin reaction**	1 (1.7%)	9 (8.8%)	0.082
**Rash**	2 (3.5%)	7 (6.8%)	0.393
**Pruritus**	0 (0%)	3 (2.9%)	0.195
**Abnormal complete blood count**	0 (0%)	10 (9.8%)	0.015
**Hepatic dysfunction**	0 (0%)	10 (9.8%)	0.015
**Thyroid dysfunction**	0 (0%)	10 (9.8%)	0.015
**Myocardial injury**	0 (0%)	5 (4.9%)	0.092
**Gastrointestinal bleeding **	3 (5.3%)	1 (0.9%)	0.094
**Dyspnea**	0 (0%)	1 (0.9%)	0.457
**Fever**	3 (5.3%)	3 (2.9%)	0.447
**Immune-associated pneumonia**	0 (0%)	1 (0.9%)	0.457
**Reduction due to AEs** Targeted drug reduction Immune drug reduction	2 (3.5%) 2 (3.5%)	3 (2.9%) 3 (2.9%) 0 (0%)	0.329
**Discontinuation due to AEs** Targeted drug discontinuation Immune drug discontinuation Targeted drug and immune drug discontinuation	5 (8.9%) 5 (8.9%)	21 (20.5%) 9 (8.8%) 3 (0.3%) 9 (8.8%)	0.005

## 4 Discussion

Hepatocellular carcinoma (HCC) is a common malignant tumour, ranking among the top of the global cancer incidence and mortality rates. Most elderly HCC patients are diagnosed at intermediate or advanced stage, and systemic therapy has become their main treatment method. However, there are few studies on systemic therapy for elderly patients, mainly from subgroup analyses of limited clinical trials, and some of the clinical trials do not include elderly patients or do not conduct age subgroup studies. Further exploration of whether first-line systemic therapy is safe and effective for elderly uHCC patients is urgently needed.

The first-line systemic treatment options for elderly uHCC patients primarily consist of targeted therapy, immunotherapy and immune combination therapy. Targeted therapies primarily function by inhibiting angiogenic pathways critical for tumor vascularization, including VEGFR, FGFR, PDGFR, etc. (20–22) Several retrospective studies of targeted therapies in elderly patients have shown that the efficacy and safety of targeted therapies in elderly patients are not significantly different from those in young patients (23–27). Immune monotherapy, represented by PD-1/PD-L1 inhibitors, is less commonly used in first-line treatment. The NCCN guidelines include Immune monotherapy (durvalumab) as the other recommended regimens for first-line treatment, rather than the preferred recommended regimen. At present, there is no study on the application of immune monotherapy in elderly patients. The combined targeted therapy and immunotherapy has become the preferred recommended regimen for uHCC, but its efficacy and safety in elderly patients have not been well-study.

We conducted a multicenter retrospective study with data from the CLEAP database including eight hospitals in China. Our study showed that elderly uHCC patients receiving combined immunotherapy have a tendency to prolong OS and PFS. The ORR in the combination immunotherapy group was 29.4%, which was significantly higher than targeted monotherapy group (3.6%, *P* = 0.001). The DCR in the combination immunotherapy group was 54.9%, and targeted monotherapy was 53.6% (*P* = 0.872). The mPFS in the targeted monotherapy and combination immunotherapy group were 7.3 and 13.2 months, respectively (*P* = 0.137), and the mOS were 16.0 and 20.0 months (*P* = 0.140). Giannini et al. (28) evaluated the prognosis of 600 untreated HCC patients. A subgroup analysis of 138 untreated patients with advanced HCC showed that the median survival time of patients over 65 years old was 8 months. In this study, mOS was greater in elderly uHCC patients than in those who did not receive treatment, regardless of whether they received targeted therapy or combination therapy. The combination immunotherapy group demonstrated potential survival advantages for elder patients, although no statistical difference was observed. This finding is consistent with the results of several RCTs and real-world studies on systemic treatment in patients with advanced HCC (17, 18, 29–31).In phase III clinical trials of IMbrave 150 (17) and CARES-310 (31), compared with the control group, the combined therapy group significantly prolonged mOS (19.2 vs. 13.4 months, 22.1 vs. 15.2 months, respectively) and mPFS (6.9 vs. 4.3 months, 5.6 vs. 3.7 months, respectively).

Subgroup analysis revealed that most subgroups benefited more from the combination immunotherapy. However, those with ascites showed greater benefit from targeted monotherapy (mPFS: 10.2 vs. 8.0 months, mOS: 15.0 vs. 10.0 months), which may be related to the mechanism of ascites formation. Malignant ascites is caused by an increased peritoneal vascular permeability. Kobold et al. (32) suggested that local vascular endothelial growth factor secretion was largely responsible for initiating and maintaining the ascitic pattern of tumor growth. If VEGF is responsible for the accumulation of liquid in the solid tumor environment, anti-VEGF therapy may not only exert anti-tumor effects but also influence the development of malignant ascites. Pichelmayer et al. (33) reported that bevacizumab is not only effective against malignant tumors but also plays a role in the symptomatic treatment of malignant ascites. Therefore, for older patients with uHCC and ascites, targeted therapy alone may enhance PFS and OS.

In correlation analyses affecting prognosis, a Cox regression model was used to analyze factors affecting OS in older patients with uHCC. Univariate analysis showed that BCLC stage C, portal vein tumor thrombus, cerebrovascular disease, surgical treatment, AFP ≥ 400 ng/mL, and high AST levels were associated with shorter OS. Multivariate analysis showed that cerebrovascular disease (HR: 0.467, 95% *CI*: 0.220–0.992, *P* = 0.048) and previous surgical treatment (HR: 0.500, 95% *CI*: 0.252–0.994, *P* = 0.048) were the independent risk factors for survival. Similar to previous studies (34, 35). These results show that cerebrovascular disease is an independent risk factor for patient survival, but no mention was made of prior surgical treatment. Our study shows that a history of surgery (such as appendectomy, percutaneous coronary intervention, splenectomy, cholecystectomy, cerebral aneurysm surgery, etc.) is also a risk factor for prognosis. Notably, the treatment regimen did not significantly affect the prognosis of older patients with uHCC.

In terms of safety, our study showed that the incidence of AEs in elderly uHCC patients in the combination immunotherapy group was higher than targeted monotherapy group. The incidence of AEs in the combination immunotherapy was 58.8%, higher compared to targeted monotherapy (44.6%). The AEs observed in the targeted monotherapy group were mainly abdominal distension, fatigue, and decreased appetite, most of which were mild and improved with adjustment and symptomatic treatment. The combination immunotherapy group experienced AEs such as fatigue, abdominal distension, hypertension, decreased appetite, and thyroid dysfunction, similar to the findings from previous studies (36, 37).In this study, the discontinuation rate due to AEs was significantly higher in the combination immunotherapy compared to the targeted monotherapy group, and the difference was statistically significant (20.5% vs. 8.9%, *P* = 0.005), indicating that while combination therapy could extend the PFS and OS of patients, it also increases the risk of AEs. In the combination immunotherapy group, nine patients (8.8%) discontinued targeted drugs, three (0.3%) discontinued immunotherapy, and another nine (8.8%) discontinued both. Although more patients discontinued targeted therapy alone than immunotherapy, this discrepancy may reflect clinical decision-making. Our results are consistent with many previous studies (38, 39) showing that combination medications may increase the occurrence of adverse effects, possibly because the synergistic effect between targeted and immunologic agents lead to an increase in the incidence of AEs (40, 41). However, the incidence of AEs was lower in the elderly patients in our study compared to RCT studies. In the age subgroup analysis of the IMbrave150, the incidence of AEs in elder patients (≥65 years) was as high as 99% in the combination group. The incidence of AEs in the combination therapy in our study was only 58.8%, and the reason may be that there is bias in follow-up, and elderly patients may not be able to distinguish their underlying diseases and AEs. In addition, due to the advanced age, decreased perception, inability to clearly describe the symptoms of discomfort, and the bias of follow-up information, whether AE is derived from immunotherapy or combined therapy increases the incidence of AEs of targeted therapy still needs to be further distinguished and discussed.

This study has some limitations. First, being retrospective, it is subject to selection bias, and the small sample size may have affected the reliability of the results. Second, the use of various targeted drugs and PD-1/PD-L1 inhibitors may have introduced variability in efficacy, influencing the results of the study.

In conclusion, targeted therapy combined with immunotherapy results in a higher ORR and longer PFS and OS in older patients with uHCC. However, patients with ascites may not benefit from this approach. In addition, it is important to note that the incidence of AEs and drug withdrawal due to adverse reactions is higher in the combination therapy group. Future large-sample, multi-center prospective clinical trials are essential to thoroughly investigate and compare the efficacy and safety of different systemic treatment strategies for older patients with uHCC.

## Data Availability

The raw data supporting the conclusions of this article will be made available by the authors, without undue reservation.
